# Biotechnological Plastic Degradation and Valorization Using Systems Metabolic Engineering

**DOI:** 10.3390/ijms242015181

**Published:** 2023-10-14

**Authors:** Ga Hyun Lee, Do-Wook Kim, Yun Hui Jin, Sang Min Kim, Eui Seok Lim, Min Ji Cha, Ja Kyong Ko, Gyeongtaek Gong, Sun-Mi Lee, Youngsoon Um, Sung Ok Han, Jung Ho Ahn

**Affiliations:** 1 Clean Energy Research Center, Korea Institute of Science and Technology, Seoul 02792, Republic of Korea; 2 Department of Biotechnology, Korea University, Seoul 02841, Republic of Korea; 3 Division of Energy and Environment Technology, KIST School, University of Science and Technology (UST), Daejeon 34113, Republic of Korea

**Keywords:** plastic waste, biodegradation, bio-upcycling, circular plastic bioeconomy, systems metabolic engineering

## Abstract

Various kinds of plastics have been developed over the past century, vastly improving the quality of life. However, the indiscriminate production and irresponsible management of plastics have led to the accumulation of plastic waste, emerging as a pressing environmental concern. To establish a clean and sustainable plastic economy, plastic recycling becomes imperative to mitigate resource depletion and replace non-eco-friendly processes, such as incineration. Although chemical and mechanical recycling technologies exist, the prevalence of composite plastics in product manufacturing complicates recycling efforts. In recent years, the biodegradation of plastics using enzymes and microorganisms has been reported, opening a new possibility for biotechnological plastic degradation and bio-upcycling. This review provides an overview of microbial strains capable of degrading various plastics, highlighting key enzymes and their role. In addition, recent advances in plastic waste valorization technology based on systems metabolic engineering are explored in detail. Finally, future perspectives on systems metabolic engineering strategies to develop a circular plastic bioeconomy are discussed.

## 1. Introduction

Plastics have become an irreplaceable material in our daily lives due to their desirable properties like corrosion resistance, durability, ease of fabrication, lightweight nature, cost-effectiveness, and transparency. Driven by high consumer demand, global plastic production reached 460 million tons in 2019 and is projected to rapidly increase to reach 1 billion tons by 2050 [1]. Due to over 150 years of effort in improving the properties of plastics, most plastics are highly resistant to natural degradation (e.g., bio-, photo-, and thermo-oxidation), requiring centuries for complete degradation [2]. In addition, only 9% of post-consumer plastics undergo recycling, while the majority end up either incinerated (12%) or disposed of in landfills (79%), due to weak plastic waste regulation/supervision, a lack of requisite infrastructure, and the collection/sorting cost of plastic waste [1]. As a result, over 4.8 billion tons of plastic waste have accumulated in poorly managed landfills, causing significant disruptions to ecosystems and directly impacting human health and social well-being [3]. Thus, it is unquestionable that environmental pollution from the accumulation of plastic waste has evolved into a grave global concern.

To address the growing issue of plastic waste accumulation, several recycling methods have been developed. Mechanical recycling, a well-established technique involving the grinding or pelletizing of plastics into small particles for reuse in new products, is perhaps the most mature technology, owing to its simplicity and intuitive approach to recycling [4]. However, post-consumer plastic waste composed of mixed plastics, non-plastic materials, and additives requires sorting and cleaning before mechanical recycling, and failure to do so often leads to down-cycling into low-quality products. Moreover, the number of times plastics can be mechanically recycled is limited as the recycled plastic deteriorates through plastic grinding or pelletizing [5]. Chemical recycling processes such as gasification, pyrolysis, and chemolysis have the capacity to break down plastics into oligomers, monomers, and gaseous products, making them suitable for handling post-consumer plastic waste [6]. However, these processes are frequently energy-intensive, utilize large quantities of chemicals, and produce greenhouse gases and toxic residues. It is expected that approximately 2.8 gigatons of CO_2_ per year will be emitted into the atmosphere by 2050 from plastic incineration [1]. Therefore, ongoing efforts are aimed at exploring alternative technologies to establish an efficient and environmentally responsible plastic waste management system, fostering a sustainable plastic economy.

**Figure 1:**
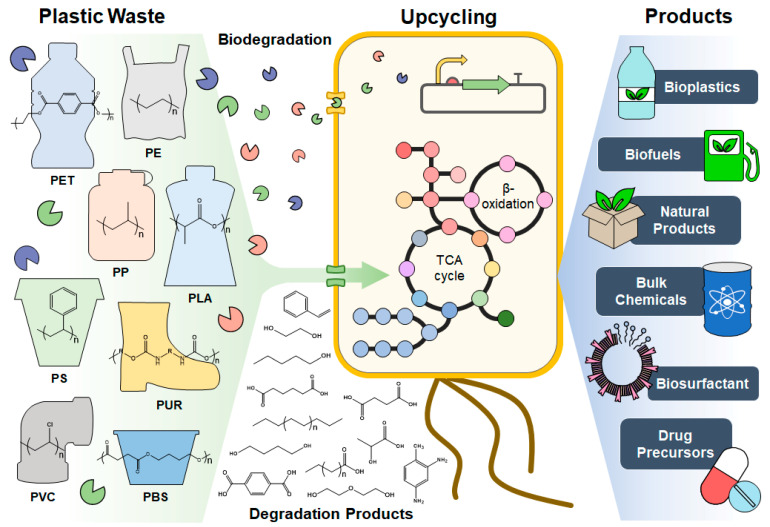
Schematic diagram of microbial and enzymatic biodegradation and bio-upcycling of plastic waste.

Microbial or enzymatic degradation of plastic waste offers a compelling alternative for plastic waste management, driven by several key advantages: (1) microorganisms have continuously evolved their metabolic capacity by creating new enzymes and extending metabolic pathways to incorporate different anthropogenic compounds (e.g., plastic) into their cellular system; (2) microorganisms and enzymes function under mild temperature and pressure conditions, without the need for toxic chemicals; (3) degradation of post-consumer plastic waste is possible without the necessity of prior sorting or cleaning. In addition, the advancements in systems metabolic engineering have accelerated the development of microbial cell factories capable of high-performance production of chemicals, fuels, and materials [7]. Hence, the degradation of plastic waste and bio-upcycling into value-added bioproducts can be integrated into a single bioprocess, which sets this approach apart from other plastic waste management technologies. This capability for biodegradation of plastic waste holds great promise in establishing a cleaner and more sustainable plastic economy (Figure 1).

The review provides a comprehensive summary of the current advances in microbial strains capable of degrading various plastics as well as key enzymes and their role in plastic degradation. Moreover, detailed information on the development of plastic waste valorization technology based on systems metabolic engineering is provided. Finally, future perspectives on establishing a circular plastic bioeconomy are discussed.

## 2. Microbial and Enzymatic Degradation of Plastics

The level of difficulty for plastic biodegradation depends on the chemical properties of the target plastics and the availability of plastic-degrading microorganisms and enzymes. Thus, the development of biodegradation technologies for different types of plastics has progressed at varying rates. Numerous microorganisms exhibiting plastic degradation capabilities have been identified in diverse environments [8]. Moreover, the degradation ability of these microorganisms/enzymes is evaluated using various methods such as the following: (1) using scanning electron microscopy (SEM), atomic force microscopy (AFM), and water contact angle (WCA) analysis to monitor the change in surface structure and hydrophobicity [9,10]; (2) using a universal mechanical testing system to assess changes in mechanical properties [11]; (3) simple weighing to investigate weight loss [12]; (4) Fourier transform infrared spectroscopy (FTIR), X-ray photoelectron spectroscopy (XPS), and nuclear magnetic resonance (NMR) to identify the formation of new functional groups [9,12]; (5) cell growth tests using plastic as a sole carbon source [10]; and (6) using high-performance liquid chromatography and gas chromatography–mass spectrometry (GC-MS) to monitor chemicals generated from plastic degradation [13]. This section summarizes the highest biodegradation performances achieved by enzymes (Table 1) and microbial strains (Table 2) for fossil-based plastics (polyethylene (PE), polypropylene (PP), polyvinyl chloride (PVC), polyurethane (PU), polyethylene terephthalate (PET), polystyrene (PS), polylactic acid (PLA), and polybutylene succinate (PBS)).

### 2.1. Fossil-Based Plastics

#### 2.1.1. Polyethylene and Polypropylene

**Figure 2:**
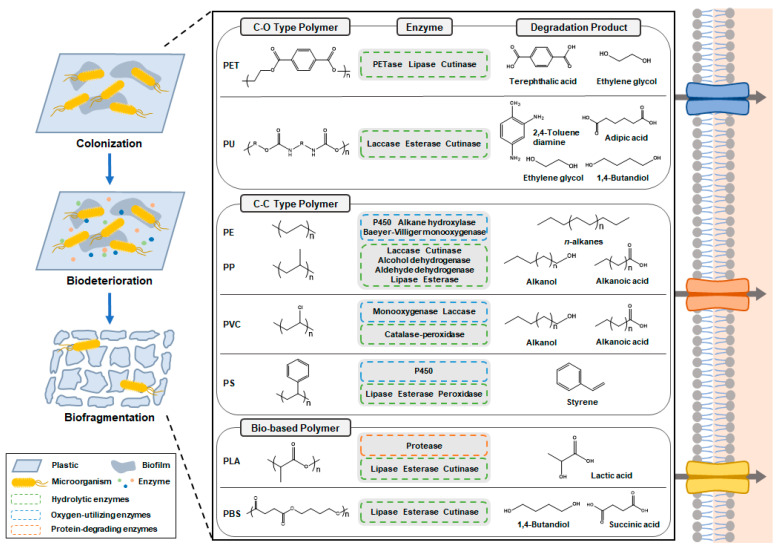
Enzymatic degradation of various types of plastics. Plastic-degrading microorganisms colonize the plastic surface and release enzymes for biodeterioration and biofragmentation.

The degradation of PE and PP proceeds at an exceptionally slow rate in the natural environment. This can be attributed primarily to the robust structural stability and hydrophobic characteristics of these polymers, owing to their backbone chains consisting exclusively of C-C and C-H bonds. Due to the challenging nature of their degradation, our understanding of the routes, mechanisms, and necessary enzymes involved in PE and PP degradation remains at an early stage. The degradation of PE and PP is envisioned to occur in three stages: (1) The first stage is the formation of hydrolyzable functional groups in the C-C backbones of PE and PP. Alkane hydroxylases [17] and cytochrome P450 [10] are potential enzymes that can hydroxylate PE and PP to initiate biodegradation (Figure 2 and Table 1) [51]. In addition, enzymes including Baeyer–Villiger monooxygenases, alcohol dehydrogenases, and aldehyde dehydrogenases can contribute to increasing the proportion of oxygen in the backbone of PE and PP [8]. (2) The second stage is the hydrolysis and fragmentation of the carbon chain. Classes of enzymes such as cutinases [14], lipases [15], and esterases [16] are potential candidates for hydrolyzing hydroxylated PE and PP into shorter-chain alkanoic acids and alkanols (Figure 2 and Table 1) [8]. These processes in steps (1) and (2) are iterated until the chain length of the degradation products of PE and PP becomes sufficiently short for microbial utilization. (3) The third stage is the assimilation of degradation products for microbial biomass formation and other metabolic pathways. While most microorganisms prefer carbon chains shorter than C10, some microorganisms, such as the *Acinetobacter* sp. M-1 strain, have been reported to utilize C13-C44 alkanes as a sole carbon source [52].

Despite the highly recalcitrant nature of PE and PP, there have been numerous reports of various bacterial, fungal, and insect species exhibiting PE degradation capabilities [8]. However, very few reports exist on successful plastic degradation to a degree where these materials can serve as a sole carbon source in metabolic pathways. In a recent study, the *Bacillus thuringiensis* JNU01 strain, isolated from a landfill site, was identified to grow from an OD600 (optical density at 600 nm) of 0.2 to 1 after 30 days in M9 minimal medium containing PE powder as a sole carbon source (Table 1) [10]. GC-MS analysis of the culture medium identified the production of PE degradation products, including C7-C29 alkanes, C9 alkene, C11 acid, C6 alcohol, and C11-C15 ethers. Moreover, FTIR, H-NMR, and XPS analyses confirmed the formation of hydroxyl, carboxyl, and amide groups within the degraded PE sample. Notably, sequence analysis and transcriptional expression profiling led to the discovery of a cytochrome P450 catalyzing the hydroxylation of PE (Table 1). In another study, the *Pseudomonas aeruginosa* WGH-6 strain, isolated from landfill soil, exhibited growth, increasing from an OD600 of 0.2 to 0.35 after 10 days when cultivated in a minimal salt medium containing PP powder as a sole carbon source (Table 2) [35]. Extended cultivation for 40 days resulted in 17.2% weight loss in the PP particles, and SEM images revealed the formation of cracks and pits on the surface of the PP particles. WCA analysis indicated a reduced contact angle (56.88°) compared to untreated PP (108.22°), confirming the PP degradation activity. Furthermore, GC-MS analysis of the culture medium identified the predominant production of C29-C35 alkanes as PP degradation products.

#### 2.1.2. Polyvinylchloride

The degradation of PVC poses significant challenges due to PVC’s hydrophobic nature and the absence of readily hydrolyzable ester bonds. As a result, the present state of research into PVC biodegradation remains in its early stages, and thus far, no specific enzymes have been identified for degrading PVC. PVC degradation is proposed to occur in four stages: (1) The first stage is the oxidative dechlorination of PVC. Enzymes such as non-heme chloroperoxidases, perhydrolases, and haloacid dehalogenase-like hydrolases have been identified as potential catalysts capable of facilitating the oxidative dechlorination of halogenated compounds [53]. (2) The second stage is the increased formation of hydrolyzable functional groups in dechlorinated PVC. Enzymes like catalase-peroxidases, monooxygenases, dioxygenases, laccases, alcohol dehydrogenases, and aldehyde dehydrogenases may play a role in enhancing the oxygen content in the PVC backbone [53]. (3) The third stage is the hydrolysis and fragmentation of oxygenated PVC. Enzymes such as esterases, dihydroxy acid dehydratases, and lipases have the potential to depolymerize oxygenated PVC into shorter compounds, including alcohols, mono-acids, and di-acids (Figure 2) [40]. (4) The fourth stage is the assimilation of degradation products into microbial biomass formation and other metabolic pathways.

Numerous studies have reported on the possibility of PVC biodegradation by various fungal and bacterial species [54,55,56]. However, there is a scarcity of reports detailing the effective degradation of PVC to a level where it can serve as a sole carbon source in metabolic pathways. In a recent study, the *Klebsiella* sp. EMBL-1 strain isolated from a larval intestine was reported to form a biofilm on the surface of a PVC film and grow from an OD600 of 0.2 to 0.6 after 10 days in a mineral salt medium (Table 2) [40]. Over the course of 90 days of cultivation, SEM images revealed the formation of cracks and pits on the PVC film surface, and 19.57% weight loss was observed in the PVC film.

GC-MS analysis identified the production of PVC degradation products, including C5-C24 alkanols, alkanoic acids, and ethers. Furthermore, FTIR and H-NMR analyses confirmed the formation of hydroxyl and ethane groups within the degraded PVC film. These findings were corroborated by WCA analysis, which indicated a reduced contact angle (76.1°) compared to untreated PVC film (86.3°). Finally, multi-omics studies and enzymatic assays identified the involvement of a catalase-peroxidase enzyme in PVC depolymerization. These findings furnish a pivotal cornerstone, offering valuable insights into the potential structure of the PVC degradation pathway.

#### 2.1.3. Polyurethane

The level of difficulty in microbial degradation of PES-PU and PE-PU varies depending on the chemical structures of these plastics. In contrast to PES-PU, the ether groups in the carbon chain of PE-PU make degradation much more difficult due to strong hydrolysis resistance [57]. Hence, most PU degradation studies have focused on PES-PU. The ester groups in the carbon chain of PES-PU make it susceptible to hydrolytic degradation [58]. PES-PU degradation using enzyme classes such as lipases, esterases, cutinases, proteases, and laccases has been reported in numerous studies (Table 1) [59]. Among those enzymes, esterases from *P. chlororaphis* [60] and *Comamonas acidovorans* TB-35 [61] which preferably hydrolyze the ester bonds in aliphatic and aromatic polyesters, releasing carboxylic acids and alcohols, have been well studied for their protein structures and molecular dynamics [62]. Few reports exist on PES-PU degradation sufficient for use in metabolic pathways as a sole carbon source. In a study, *C. acidovorans* TB-35 isolated from soil was reported to grow up to an OD580 of 2.48 after 7 days in a mineral salt medium while consuming 50 mg of PES-PU as a sole carbon source (Table 2) [41]. The main PES-PU degradation products are composed of adipic acid, 1,4-butanediol (1,4-BDO), 2,4-toluenediamine (2,4-TDA), and ethylene glycol (EG) (Figure 2) [63].

The soft segments of PES-PU, corresponding to the polyol part, are predicted to biodegrade after the oxidation of the α-methylene hydrogen atom in polyether. However, no enzymes that degrade the soft segment of PES-PU have been reported up to now. The hard segments of PES-PU, corresponding to the isocyanate part, are reported to be degraded by *Staphylococcus epidermis* urease via urea bond hydrolysis [64]. In another study, the *Rhodococcus equi* TB-60 strain isolated from soil was reported to grow in a minimal salt medium containing toluene-2,4-dicarbamic acid dibutyl ester (TDCB), a compound with a chemical structure similar to PES-PU [42]. This bacterium degraded 2.1 mM of TDCB in 10 days and was identified to produce 2,4-TDA and carbamic acid butyl ester as degradation products using GC-MS. Furthermore, urethane hydrolase from the *R. equi* TB-60 strain was determined to hydrolyze aliphatic and aromatic urethane compounds (Table 2).

#### 2.1.4. Polyethylene Terephthalate

The identification of PET-degrading microorganisms, including *Thermobifida fusca*, *Fusarium solani pisi*, and *Ideonella sakaiensis*, has played a substantial role in advancing the field of PET biodegradation and has paved the way for PET recycling [65]. Since the initial discovery of these microorganisms, numerous native enzymes have been identified and further engineered to enhance the efficiency of PET degradation. PET is known to degrade through the following pathway: (1) PET hydrolyzes to bis(2-hydroxyethyl) terephthalate (BHET), mono(2-hydroxyethyl) terephthalate (MHET), TPA, and EG. Enzymes classes such as PETases, cutinases, leaf-branch compost cutinase (LCC), lipases, hydrolases, and esterases are known to hydrolyze PET (Table 1) [22,23,66]. Among these enzymes, a recent study applied rational engineering techniques, such as molecular docking and the analysis of enzyme contact surfaces, to enhance the catalytic activity of LCC [23]. The engineered LCC, with residue changes at F243W/D238C/S283C/Y127G, exhibited exceptional PET degradation capability, producing TPA at a rate of 16.7 g/L/h using 3 mg of enzymes per g of PET, representing the highest reported performance in PET degradation to date. (2) BHETase hydrolyzes BHET to MHET, TPA, and EG. Until recently, heterogeneous degradation products (MHET, BHET, TPA, EG) were obtained from PET degradation due to the absence of BHETase. This resulted in reduced product yield and compromised physical properties in recycled PET synthesis [67]. However, a recent study identified two BHETases from the *B. subtilis* PET-86 and *Chryseobacterium* sp. PET-29 strains capable of hydrolyzing BHET (Table 1) [26]. This discovery of BHETase completes the PET biodegradation pathway and opens the possibility for fully bio-based PET recycling. (3) MHET is hydrolyzed to TPA and EG. While the enzymes described in step (1) have little effect on MHET hydrolysis, MHETase derived from *I. sakaiensis* has been identified to hydrolyze MHET with high substrate specificity (Table 1) [43]. Given its crucial role in complete PET degradation, extensive studies have been conducted on the crystal structure and catalytic mechanism of MHETase [68]. These findings have paved the way for substantial efforts in protein engineering of MHETase to enhance its catalytic performance and enable the co-utilization of MHET and BHET as substrates [27]. (4) TPA and EG are assimilated into microbial biomass formation and other metabolic pathways. A study reported that both the *P. putida* KT2440 and JM37 strains can grow using EG as a sole carbon source [69]. These strains of *P. putida* have the ability to convert EG into glyoxylate, which is then incorporated into the central carbon network to support biomass formation. Furthermore, another study unveiled the discovery of the novel *Comamonas* sp. E6 strain, which possesses the capability to grow using TPA as the sole carbon source [70]. This finding marks the first instance of a strain with such a unique ability. The *Comamonas* sp. E6 strain possesses the ability to convert TPA into protocatechuic acid (PCA), which subsequently enters the PCA 4,5-cleavage pathway, contributing to biomass synthesis.

#### 2.1.5. Polystyrene

While the complete degradation mechanisms and pathways of PS remain a subject of ongoing research, degradation pathways have been proposed based on studies involving PS-degrading microorganisms (Table 2) [44,45]: (1) The first is the cleavage of the main chain of PS into aromatic monomers, such as styrene. Enzymes such as lipases, esterases, P450, and peroxidases are hypothesized to cleave the main chain of PS (Table 1) [71]. (2) The second is the conversion of styrene into acetyl-CoA. (3) The third is the utilization of acetyl-CoA in the tricarboxylic acid (TCA) cycle for microbial biomass formation and other metabolic pathways.

Until recently, there have been no reports on the degradation of PS mediated by microorganisms. In a study, the *P. aeruginosa* DSM 50071 strain isolated from the gut of *Zophobas atratus*, a Styrofoam-eating larva, was identified for the first time to grow in a carbon-free basal solid medium using PS as a sole carbon source (Table 2) [44]. After 60 days of cultivation, the SEM image of the PS particle surface showed edge smoothing and the formation of holes. These findings were consistent with the results of WCA analysis, indicating a reduced contact angle of 79.8° compared to untreated PS (91.56°). Moreover, XPS and FTIR analyses revealed the formation of hydroxyl and carbonyl groups, as well as a reduction in C-C bonds, providing evidence for PS biodegradation. In another study, the *A. johnsoniii* JNU01 strain, isolated from soil, exhibited PS-concentration-dependent growth in a basal salt medium utilizing PS powder (Table 2) [45]. Most importantly, a whole genome sequence and transcriptional analyses of *A. johnsoniii* JNU01 uncovered an alkane-1-monooxygenase responsible for catalyzing PS hydroxylation.

### 2.2. Bio-Based Plastics

#### 2.2.1. Polylactic Acid

While PLA is considered a biodegradable plastic, only 24 enzymes associated with PLA biodegradation have been extensively characterized until now [72]. PLA undergoes degradation through enzymatic hydrolysis of its ester bonds, followed by the assimilation and metabolism of lactic acid oligomers, dimers, and monomers. PLA biodegradation is primarily facilitated by enzymes such as proteases, lipases, esterases, and cutinases, mainly found in genera such as *Amycolatopsis*, *Alcanivorax*, *Bacillus*, and *Pseudomonas* (Table 1) [30,31,73].

Numerous microorganisms have demonstrated the capability to degrade PLA, and one such strain is *Saccharothrix waywayandensis*, which was isolated from soil. In a study, this microbial strain was reported to grow up to 37 mg of DCW when cultivated in a basal medium containing yeast extract and 100 mg of PLA film as the carbon sources (Table 2) [47]. In addition, this strain degraded 95% of PLA film within 7 days with the supplementation of 0.1% (*w*/*v*) gelatin, a well-known inducer of the hydrolases previously elucidated (Table 2) [74,75]. The supplementation of gelatin proved to be highly effective in augmenting the expression levels of PLA-degrading hydrolases, thereby promoting a more efficient degradation process. Furthermore, during the course of PLA degradation, a transient increase in lactic acid generation was observed. Nonetheless, this temporary elevation in lactic acid levels was reduced as the strain assimilated the generated lactic acid.

#### 2.2.2. Polybutylene Succinate

The degradation mechanism of PBS, along with PBS-degrading microorganisms and enzymes, is well established. PBS is known to undergo degradation through hydrolytic cleavage of the ester bonds, catalyzed by extracellular enzymes such as lipases and cutinases (Table 1) [32,76]. These hydrolytic enzymes break down PBS into various components, including oligomers (bis(4-hydroxybutyl) succinic acid and 4-(4-((3-carboxypropanoyl)oxy)butoxy)-4-oxobutanoic acid), a dimer (4-(4-hydroxybutoxy)-4-oxobutanoic acid), and monomers (succinic acid and 1,4-BDO) [77]. Furthermore, the oligomers and dimers resulting from PBS degradation are fully hydrolyzed into succinic acid and 1,4-BDO monomers, which then enter the central carbon metabolism.

Currently, PBS-degrading enzymes such as lipase Asahi from *Chromobacterium viscosum* [76] and lipase PS^®^ from *P. cepacia* [34] are commercially available (Table 1). PBS-degrading microorganisms have predominantly been identified among fungi [32] and microbial consortia [78]. However, there is a need to explore other varieties of microorganisms to establish more efficient PBS degradation and bio-upcycling technologies. In a recent study, the *Bacillus* sp. JY35 strain was isolated from wastewater sludge, demonstrating its ability to degrade a wide range of bioplastics, including PBS, PHA, and polycaprolactone [79]. The whole genome sequencing of this strain revealed the presence of a carboxylesterase capable of degrading PBS. To explore the potential of this enzyme, an engineered *Escherichia coli* strain was developed to overexpress the *Bacillus* sp. JY35 carboxylesterase. The engineered *E. coli* strain exhibited time-dependent PBS degradation, effectively breaking down 45.7% of 20 mg of PBS over a period of 10 days (Table 2).

## 3. Valorization of Plastic Degradation Products into Useful Chemicals and Materials

As described in the previous sections, plastic-degrading microorganisms release extracellular enzymes that break down plastics into various acids, alcohols, and carbon-ring compounds (Figure 2 and Table 1). These compounds can serve as substrates for biomass formation and for the production of diverse and valuable bio-based products [80].

The discovery of microbial strains capable of utilizing plastic waste has demonstrated the feasibility of employing plastic waste as an alternative substrate to fuel central carbon metabolism [81]. Nonetheless, natural metabolic pathways are not the exclusive options. Recent progress in systems metabolic engineering and genome editing technologies has facilitated the incorporation of exogenous pathways or the construction of entirely novel metabolic routes [3]. Significant scientific investigations are currently in progress to explore the bio-upcycling of these plastic wastes into a range of valuable chemicals, including surfactants, important metabolites, and several types of biopolymers, with the ultimate goal of establishing a circular plastic economy [82]. This section explores the systems metabolic engineering strategies for developing microbial strains capable of producing value-added chemicals and materials using the plastic degradation products (hexadecanoic acid, lactic acid, 1,4-BDO, EG, styrene, adipic acid, and TPA) outlined in Section 2 as substrates.

### 3.1. Hexadecan(oat)e

**Figure 3:**
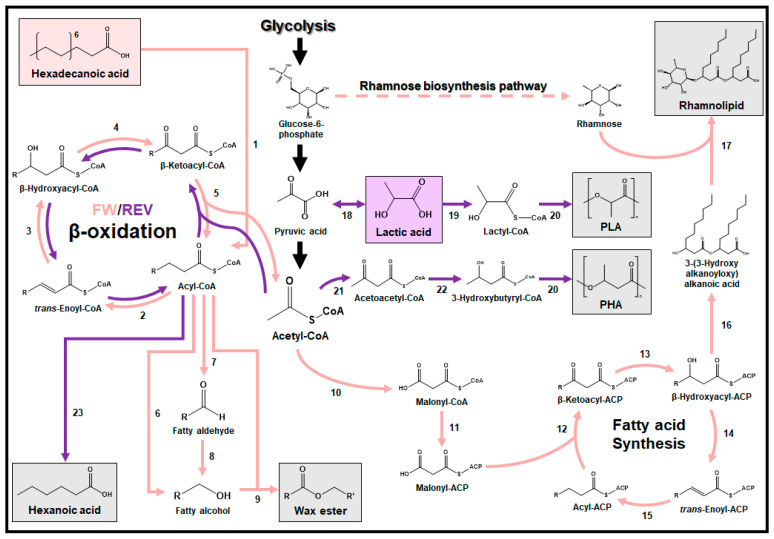
Metabolic pathways for bio-upcycling of hexadecanoic and lactic acids. Each metabolic pathway for bio-upcycling is indicated by the color of the respective substrate box. Single reactions are denoted with a solid line, while multiple reactions are represented with a dashed line. 1: acyl-CoA synthetase; 2: acyl-CoA dehydrogenase; 3: enoyl-CoA hydratase; 4: hydroxyacyl-CoA dehydrogenase; 5: ketoacyl-CoA thiolase; 6: alcohol-forming fatty acyl-CoA reductase; 7: aldehyde-forming fatty acyl-CoA reductase; 8: fatty aldehyde reductase; 9: wax ester synthase/acyl-CoA:diacylglycerol acyltransferase; 10: acetyl-CoA carboxylase; 11. malonyl-CoA transacylase; 12: β-ketoacyl ACP synthase; 13: β-ketoacyl ACP reductase; 14: β-hydroxyacyl-ACP dehydrase; 15: enoyl-ACP reductase; 16: 3-(3-hydroxyalkanoyloxy)alkanoate (HAA) synthase; 17: rhamnosyltransferase; 18: lactate dehydrogenase; 19: propionyl-CoA transferases; 20: PHA synthase; 21: β-ketothiolase; 22: acetoacetyl-CoA reductase; 23: acetate-CoA transferase.

Hexadecanoic acid (also known as palmitic acid), along with other alkanoic acids, can play a pivotal role in two essential processes within microorganisms: (1) lipid production [83] and (2) fatty acid synthesis and degradation (Figure 3) [84]. Wax esters, valuable lipids extensively used in the cosmetic industry, can be synthesized from hexadecanoic acid through the lipid synthesis pathway (Figure 3). In a recent study, a high-density PE (HDPE) valorization process that combines chemical catalysis with bioconversion using microbial consortia to produce wax esters was developed [85]. The microbial consortia, obtained from plastic-enriched soil, demonstrated the ability to produce various wax esters, including lauryl palmitate, myristyl palmitate, and cetyl palmitate. These wax esters were generated using hexadecanoic acid and C12-C16 alkanols obtained from the catalytic deconstruction of HDPE. It should be noted that alkanols and alkanoic acids are known to enter the cytosol through various mechanisms, including transporters [86], efflux pumps [87], and passive diffusion across the lipid bilayer [88]. Furthermore, *R. aetherivorans* was identified as the main player in wax ester production through whole-gene 16S rDNA sequencing of the microbial consortia and cultivation tests using HDPE degradation products [85]. *Rhodococcus* species are known to synthesize wax esters through the esterification of alkanols and acyl-CoA, both of which can originate from hexadecanoic acid, facilitated by the enzyme wax ester synthase/acyl-CoA:diacylglycerol acyltransferase [89]. Alkanols required for wax ester production can be synthesized through either a single enzymatic reaction, involving alcohol-forming fatty acyl-CoA reductase, or a sequential enzymatic reaction involving aldehyde-forming fatty acyl-CoA reductase and fatty aldehyde reductase. High concentrations of alkanols can damage the cell membrane by denaturing and coagulating membrane proteins, leading to a reduction in cell growth [90,91]. Similarly, high concentrations of alkanoic acids are known to disrupt the cell membrane, inhibit protein synthesis, and impede cell wall biosynthesis [92].

Rhamnolipid, a biosurfactant used in cosmetic and medical applications, can be synthesized from hexadecanoic acid via the fatty acid synthesis, β-oxidation, and rhamnose production pathways. In a particular study, the *Dietzia maris* As-13-3 strain, isolated from a deep-sea hydrothermal field, demonstrated the ability to produce 120 mg/L of rhamnolipid from a 2% (*v*/*v*) hexadecane substrate (Figure 3) [93]. Hexadecane along with other *n*-alkanes are plastic degradation products that are converted to fatty acids (i.e., hexadecanol) for degradation [3]. The rhamnolipid biosynthesis pathway within the *D. maris* As-13-3 strain was fully elucidated through genome sequencing. Within this biosynthesis pathway, hexadecanoic acid, derived from hexadecane through the action of alcohol/aldehyde dehydrogenases, undergoes conversion into hexadecanoyl-CoA via acyl-CoA synthetase. The hexadecanoyl-CoA molecule enters the β-oxidation pathway, breaking down into acetyl-CoA, which further participates in the fatty acid synthesis pathway to yield two molecules of β-hydroxyacyl-ACP for the production of 3-(3-hydroxyalkanoyloxy) alkanoic acid (HAA). Finally, rhamnose, produced through the rhamnose production pathway, combines with HAA catalyzed by rhamnosyltransferase (RhlB) to produce rhamnolipid. Quantitative real-time PCR analysis of the *D. maris* As-13-3 strain grown on hexadecane revealed that HAA synthase and RhlB are pivotal enzymes for rhamnolipid production. This result lays the groundwork for future studies in systems metabolic engineering of microbial strains aimed at enhancing rhamnolipid production from hexadecanoic acid.

### 3.2. Lactic Acid

Numerous studies have reported the existence of microorganisms with the capability to produce various-chain-length fatty acids while co-utilizing lactic acid and sugar as substrates [94]. Among the fatty acids that can be produced using microorganisms, hexanoic acid serves as a versatile platform chemical with a broad spectrum of applications encompassing antimicrobial agents, lubricants, fragrances, pharmaceuticals, and biofuels [7]. In particular, *Megasphaera hexanoica*, isolated from a cow rumen, was recently found to produce 8.9 g/L of hexanoic acid using the reverse β-oxidation pathway when provided with 10 g/L of lactic acid as the sole electron donor (Figure 3) [95]. Furthermore, the co-utilization of lactic acid and fructose as dual electron donors not only enhanced cell growth but also resulted in the production of 13.8 g/L of hexanoic acid with a maximum productivity of 20.9 g/L/day. This achievement represents the highest hexanoic acid production to date. In another study, the discovery of a repressor gene responsible for inhibiting lactic acid utilization in the presence of sugar was made within this hexanoic acid-producing microorganism. Understanding and addressing this regulatory network will be essential for enhancing fatty acid production from lactic acid [94]. It is noteworthy that lactic acid easily crosses the cell membrane via passive diffusion, and this inherent property has the potential to induce alterations in cytosolic pH and perturbations in the anion pool, thereby exerting inhibitory effects on microbial growth [96].

There have been significant efforts in developing microbial strains for the production of biodegradable lactic acid-containing polyesters [97,98]. To establish a metabolic pathway for the production of these polyesters, it is essential to incorporate both the lactyl-CoA production pathway and PHA synthase (PhaC), capable of utilizing lactyl-CoA as a substrate. In a specific study, *Clostridium propionicum* propionyl-CoA transferases (Pct) and *Pseudomonas* sp. 61-3 PhaC were introduced into the *E. coli* XL1-Blue strain to develop a strain capable of producing PLA from lactic acid (Figure 3) [99]. Moreover, by further incorporating *Cupriavidus necator* β-ketothiolase (PhaA) and acetoacetyl-CoA reductase (PhaB) into another strain, a strain capable of producing poly(3HB-co-LA) was developed (Figure 3). In order to improve PLA and poly(3HB-co-LA) production, random mutagenesis of Pct was conducted via mutagenic PCR, resulting in a mutant Pct (V193A, T78C, T669C, A1125G, and T1158C) capable of efficiently converting lactate to lactyl-CoA. Furthermore, saturation and site-directed mutagenesis were performed on PhaC, leading to the development of a mutant PhaC (E130D, S325T, and Q481M) that could effectively accept lactyl-CoA as a substrate. In a subsequent study, the *E. coli* XL1-Blue strain, equipped with the mutant Pct and PhaC and native PhaAB, underwent metabolic engineering based on in silico genome-scale metabolic flux analysis and flux response analysis, resulting in an increased production of PLA and poly(3HB-co-LA) [100]. This engineering process commenced with the deletion of the gene responsible for acetate kinase and the replacement of the native promoter of the gene encoding acetyl-CoA synthetase with a strong *trc* promoter. These modifications aimed to augment the acetyl-CoA pool, which serves as a vital CoA donor for lactyl-CoA regeneration. Additionally, the native promoter of the gene encoding lactate dehydrogenase was exchanged with a *trc* promoter, and genes encoding phosphoenolpyruvate carboxylase and acetaldehyde/alcohol dehydrogenase were deleted to redirect the metabolic flux toward lactyl-CoA. Employing the final engineered strain, PLA and poly(3HB-co-LA) were produced at levels of up to 11% (*w*/*w*) and 46% (*w*/*w*) of dried cell weight (DCW), respectively. GC analysis confirmed that a molar yield of up to 70% of lactic acid was integrated into the poly(3HB-co-LA) production.

### 3.3. 1,4-Butanediol

**Figure 4:**
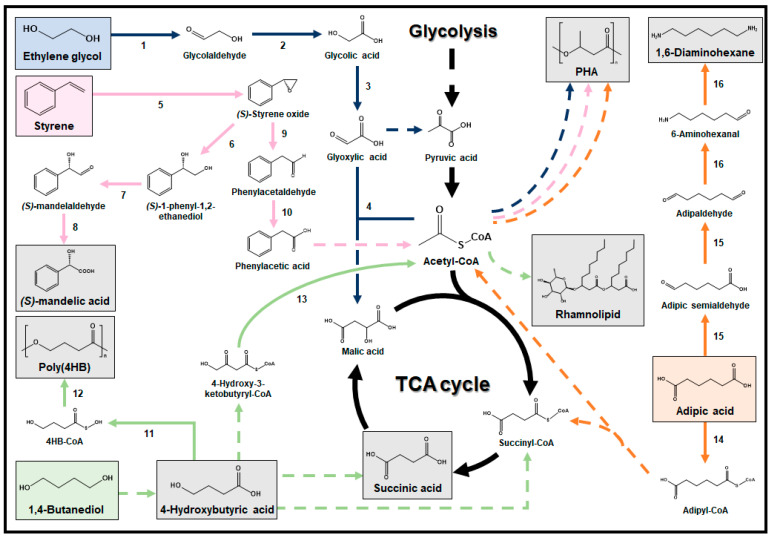
Metabolic pathways for bio-upcycling of ethylene glycol (EG), styrene, 1,4-butanediol (1,4-BDO), and adipic acid. Each metabolic pathway for bio-upcycling is indicated by the color of the respective substrate box. Single reactions are denoted with a solid line, while multiple reactions are represented with a dashed line. 1: glycoaldehyde reductase; 2: glycolaldehyde dehydrogenase; 3: glycolate oxidase; 4: malate synthase; 5: styrene monooxygenase; 6: epoxide hydrolase; 7: alcohol dehydrogenase; 8: phenylacetaldehyde dehydrogenase; 9: styrene oxide isomerase; 10: phenylacetaldehyde dehydrogenase; 11: 4HB-CoA:CoA transferase; 12: PHA synthase; 13: acetyl-CoA acetyltransferase; 14: adipyl-CoA transferase; 15: carboxylic acid reductase; 16: transaminase.

1,4-BDO, previously confirmed as non-toxic to microorganisms, holds significant value as a substrate for the production of a wide range of platform chemicals and polymers using systems metabolic engineering in microorganisms [101]. Succinic acid, an important building block chemical used across diverse industries including food, pharmaceuticals, petrochemicals, and plastics, can be derived from 1,4-BDO (Figure 4) [102,103]. In a recent study, the *P. putida* NB10 strain was isolated using atmospheric pressure room-temperature plasma-induced mutation integrated with adaptive laboratory evolution (ALE) to improve 1,4-BDO utilization and synthesize succinic acid through multiple oxidoreductases [104]. It should be noted that *P. putida* utilizes a polyamine ABC transporter to transfer 1,4-BDO into the cytosol [103]. To further enhance succinic acid production from 1,4-BDO, the deletion of the *sdhABCD* operon, which encodes succinate dehydrogenase, can prevent the conversion of succinic acid within the TCA cycle [105]. Moreover, addressing intracellular succinic acid accumulation, which hampers cell growth, can be achieved by enhancing succinic acid transport through heterologous expression or overexpression of succinate transporters [106].

4-hydroxybutryic acid (4HB), a precursor to a range of industrial chemicals and polymers including 4-butyrolactone and various PHAs, is another platform chemical that can be produced from 1,4-BDO via sequential reactions involving alcohol dehydrogenase, aldehyde dehydrogenase, and oxidoreductase (Figure 4) [103,107]. To enhance 4HB accumulation, the deletion of the *sad* and *puuE* genes, which encode succinate semialdehyde dehydrogenase and aminotransferase, respectively, can prevent 4HB consumption [107]. Furthermore, the strategy for 4HB production from 1,4-BDO can be extended to the synthesis of poly(4HB), a biodegradable polymer widely employed in various biomedical applications (Figure 4) [108]. In a study that focused on poly(4HB) production, the poly(4HB) production pathway was constructed within the *E. coli* JM109 strain by introducing two key enzymes: 4HB-CoA:CoA transferase from *C. kluyveri* and PhaC from *Ralstonia eutropha* [108]. Notably, this engineered strain achieved the production of poly(4HB) at levels reaching up to 65% (*w*/*w*) of the DCW. The deletion of acetate kinase and overexpressing acetyl-CoA synthetase as a strategy to increase the acetyl-CoA pool for enhanced PHA production has been employed in previous studies [109].

Rhamnolipid is also a valuable compound produced from 1,4-BDO using microorganisms (Figure 4). In one study, an engineered strain of *P. putida* was developed through ALE, enabling it to produce rhamnolipid using 1,4-BDO as a sole carbon source [110]. Although the rhamnolipid production pathway from 1,4-BDO has not been fully elucidated, the engineered strain produced 0.13 g/L of rhamnolipid with a yield of 0.09 g/g from 1,4-BDO. This production process is hypothesized to involve the conversion of 4HB, originating from 1,4-BDO, into 4-hydroxy-3-keto-butyryl-CoA through a sequence of enzymatic reactions, including acyl-CoA dehydrogenase, enoyl-CoA hydratase, and 3-hydroxyacyl-CoA dehydrogenase [103]. Subsequently, acetyl-CoA acetyltransferase cleaves 4-hydroxy-3-keto-butyryl-CoA into glycolyl-CoA and acetyl-CoA, and acetyl-CoA is then utilized in the production of rhamnose through the same pathway as described in Section 3.1 (Figure 3).

### 3.4. Ethylene Glycol

Several microorganisms capable of EG metabolism have been reported, with *Pseudomonas* being a well-studied example [111]. In one study, the *P. putida* KT2440 strain was metabolically engineered to efficiently utilize EG and produce medium-chain-length PHA from EG (Figure 4) [69]. First, the operon associated with the glyoxylate carboligase (gcl) pathway, consisting of genes encoding gcl, hydroxypyruvate isomerase, tartronate semialdehyde reductase, hydroxypyruvate reductase, and pyruvate kinase, was overexpressed by replacing its native promoter in the genome with a constitutive *tac* promoter. This modification allowed for cell growth on minimal medium supplemented with EG as a sole carbon source. However, it was observed that there was a significant accumulation of glycolic acid, suggesting a low conversion rate of glycolic acid to glyoxylic acid. Additionally, toxicity assays using various metabolites involved in the gcl pathway indicated that glycolaldehyde was the key intermediate inhibiting the growth of *P. putida* on EG. To address this issue, the operon encoding glycolate oxidase was overexpressed by replacing its native promoter with a constitutive *tac* promoter, thus preventing the accumulation of glycolaldehyde and glycolic acid. The overexpression of both gcl and glycolate oxidase facilitated the growth of the engineered strain even in EG concentrations as high as 2 M, with the consumption of 500 mM of EG achieved within 120 h. Notably, a separate toxicity test of EG in *E. coli*, conducted in a different study, revealed that a concentration as high as 4.5 M of EG completely inhibits cell growth [112]. Moreover, since the *P. putida* KT2440 strain inherently possesses the PHA production pathway, the final engineered strain produced 32.19% of DCW as medium-chain-length PHA with a yield of 0.06 g/g from EG under nitrogen-limited conditions.

While most reported EG bio-upcycling studies have focused on PHA production, it is anticipated that a wider range of EG bio-upcycling products will come into play [111]. This expectation stems from the EG metabolic pathway’s ability to generate crucial intermediates like glyoxylic acid, pyruvic acid, and acetyl-CoA, all of which are closely linked to central carbon metabolism, theoretically allowing for the production of various related products (Figure 4). Furthermore, the small molecular size of EG enables it to easily cross the cell membrane via passive diffusion [113].

### 3.5. Styrene

Styrene can serve as a substrate for the production of aromatic compounds and biopolymers through systems metabolic engineering of microorganisms. A modular cascade biocatalyst was developed to enable the regio- and enantioselective oxy-functionalization of styrene, leading to the production of mandelic acid. Mandelic acid is widely used in the cosmetic and pharmaceutical industries for skincare applications. In this study, two enzyme modules were designed, handling (1) the conversion of styrene to *(S)*-1-phenyl-1,2-ethanediol and (2) the conversion of *(S)*-1-phenyl-1,2-ethanediol to *(S)*-mandelic acid, based on biocatalytic retrosynthesis analysis (Figure 4) [114]. The first module was implemented in *E. coli* by introducing *Pseudomonas* sp. VLB120 styrene monooxygenase and *Sphingomonas* sp. HXN-200 epoxide hydrolase. Furthermore, the second module was constructed by introducing a highly regioselective *P. putida* GPo1 alcohol dehydrogenase and the native phenylacetaldehyde dehydrogenase. Finally, a dual-phase biotransformation process was conducted using the final engineered strain carrying both modules [115]. This involved achieving a high cell density during the growth phase, followed by introducing styrene dissolved in a biocompatible organic solvent, specifically biodiesel, for the *(S)*-mandelic acid production phase. As a result, 20.7 g/L of *(S)*-mandelic acid was produced with a molar yield of 68% from styrene within 29 h [116]. It should be noted that aromatic compounds, including styrene, can impair cell membrane function even when present in low concentrations [117].

In another study, *Enterobacter* spp. isolated from diesel-contaminated soil were identified to grow and produce PHA using styrene as a sole carbon source [118]. While the mechanism of styrene uptake into the cytosol and the precise PHA production pathway from styrene remain unelucidated, a proposed styrene metabolic pathway suggests that styrene monooxygenase catalyzes the oxidation of styrene to styrene oxide, which is then isomerized to phenylacetaldehyde by styrene oxide isomerase (Figure 4) [119]. Subsequently, phenylacetaldehyde is further metabolized by phenylacetaldehyde dehydrogenase to yield phenylacetic acid. This compound undergoes additional metabolic processes, with the specific enzymes involved still unidentified, resulting in its conversion into acetyl-CoA. Finally, the acetyl-CoA molecule serves as a precursor for the production of PHA using the same pathway as described in Section 3.2 (Figure 3).

### 3.6. Adipic Acid

Adipic acid, previously established as toxic to microorganisms only at high concentrations (>650 mM), can serve as a valuable substrate for the production of various platform chemicals and polymers when leveraging systems metabolic engineering in microorganisms [120]. In one study, systems metabolic engineering was applied to the *P. putida* KT2440 strain to enable the production of PHA from adipic acid (Figure 4) [121]. To establish the adipic acid metabolic pathway in *P. putida*, the *dcaAKIJP* operon from *A. baylyi*, which encodes adipyl-CoA transferase, dehydrogenase, and putative adipic acid uptake proteins, was initially integrated into the genome. However, it was observed that the engineered strain containing the *dcaAKIJP* operon could not grow on adipic acid. This limitation may be attributed to insufficient induction of the native genes responsible for the downstream 2,3-didehydroadipyl-CoA metabolic pathway. In order to develop a strain capable of growing on adipic acid as a sole carbon source, ALE involving a stepwise increase in adipic acid feeding while reducing the supply of glucose was conducted. Genome sequencing of the evolved strain revealed that expression of the *paa* gene cluster associated with the phenylacetate degradation pathway improves adipic acid metabolism [121]. Moreover, the regulator-encoding *psrA* gene was identified to upregulate the redundant β-oxidation genes. Based on these findings, rational reverse engineering of the evolved strain was performed, involving the overexpression and removal of the *paa* cluster and *psrA* genes, respectively. As a result, the engineered *P. putida* strain was able to utilize adipic acid, achieving a biomass concentration of 0.71 g DCW per liter. Furthermore, the engineered strain produced PHA at a level of up to 25.3% of the biomass under nitrogen-limited conditions using the same pathway described in Section 3.2 (Figure 3).

In another study, the *E. coli* BL21 strain was metabolically engineered to produce 1,6-diaminohexane, a monomer of polyamide-6/6, from adipic acid (Figure 4). To accomplish this, a synthetic 1,6-diaminohexane production pathway was established in *E. coli*, employing two carboxylic acid reductases and transaminases [122]. Enzyme mining determined that a sequence of reactions involving carboxylic acid reductases from *Mycolicibacterium smegmatis* MC2 155 and *Mycobacteroides abscessus*, along with transaminases from *Silicibacter pomeroyi* and *E. coli*, constituted the optimal enzyme combination for effective 1,6-diaminohexane production. Subsequently, the expression levels of the four enzymes in the 1,6-diaminohexane pathway were fine-tuned by assessing their impact on 1,6-diaminohexane production using vectors with various copy numbers. In addition, as both carboxylic acid reductases are NADPH-dependent, two transhydrogenase genes (*sthA* and *pntAB*) and the *pgi* and *pfkA* genes encoding glucose 6-phosphate isomerase and 6-phosphofructokinase, respectively, were deleted from the genome to increase the supply of NADPH. As a result, the engineered *E. coli* strain produced 238.5 mg/L of 1,6-diaminohexane with a yield of 0.04 g/g from adipic acid.

### 3.7. Terephthalic Acid

**Figure 5:**
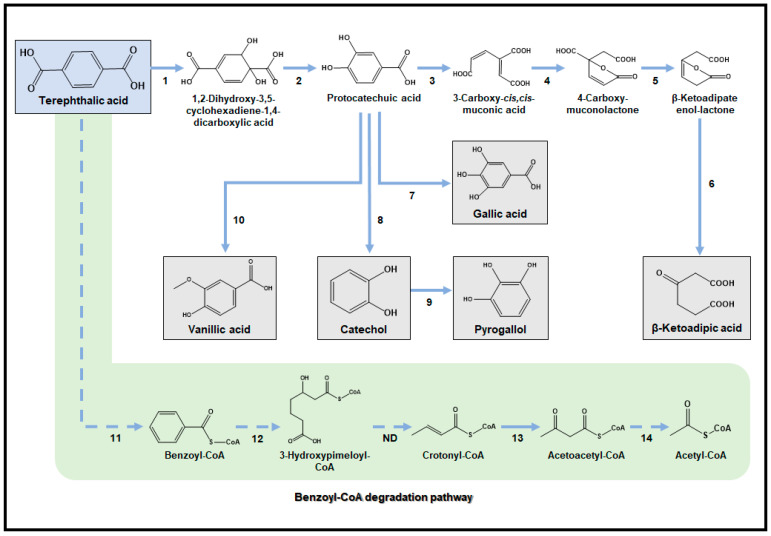
Metabolic pathways for bio-upcycling of TPA. Single reactions are denoted with a solid line, while multiple reactions are represented with a dashed line. 1: TPA dioxygenase; 2: DCD dehydrogenase; 3: PCA 3,4-dioxygenase; 4: 3-carboxy-*cis*,*cis*-muconate cycloisomerase; 5: 4-carboxymuconolactone decarboxylase; 6: 3-oxoadipate enol-lactonase; 7: *p*-hydroxybenzoate hydroxylase; 8: PCA decarboxylase; 9: catechol hydrolase; 10: O-methyltransferase; 11: TPA decarboxylase and benzoic acid CoA ligase; 12: cyclohexa-1,5-dienecarbonyl-CoA hydratase, 6-hydroxycyclohex-1-ene-1-carboxyl-CoA dehydrogenase, 6-oxo-cyclohex-1-ene-carbonyl-CoA hydrolase; 13: crotonase; 14: β-hydroxybutyryl-CoA dehydrogenase, acetyl-CoA acetyltransferase; ND: not determined.

TPA has the potential to act as a substrate for generating diverse aromatic compounds (gallic acid (GA), pyrogallol (PYG), and vanillic acid (VA)), which are often challenging to produce from traditional substrates like glucose. Among these aromatic compounds, GA, a key component used in the synthesis of anti-neoplastic drugs [123] and antioxidants [124] within the pharmaceutical industry, has been reported to be produced using TPA by an engineered *E. coli* (Figure 5) [125]. It should be noted that a high concentration of TPA has been shown to inhibit the growth of microorganisms, with concentrations reaching 60 g/L in *E. coli* and 90 g/L in *P. putida* KT2440 [126]. As PCA is an important precursor for GA production, an engineered *E. coli* strain capable of producing PCA from TPA was initially developed by introducing two key enzymes, TPA 1,2-dioxygenase and 1,2-dihydroxy-3,5-cyclohexadiene-1,4-dicarboxylate dehydrogenase, sourced from the *Comamonas* sp. E6 strain. Those key enzymes have the advantage of utilizing both NADH and NADPH as cofactors [70]. In addition, the introduction of *P. putida* KT2440 *p*-hydroxybenzoate hydroxylase (PobA), capable of hydroxylating the meta position of PCA, into the PCA-producing strain facilitated the conversion of PCA into GA. To further enhance hydroxylation activity, a molecular docking simulation of PobA was performed to gather insights for structure-based enzyme engineering. This simulation indicated that reducing the binding distance between PCA and flavin adenine nucleotide in the active site could be beneficial. Based on this finding, a final engineered strain was developed, incorporating a mutant PobA (T294A, Y385F), which produced 2.5 mM of GA from PCA. This achievement resulted in a molar yield of 74.3% in 12 h, which was an 83.7% increase compared to the strain expressing the wild-type PobA [125].

In the same study, the PCA-producing strain was further engineered to develop a whole-cell bioconversion system for the production of PYG, an antioxidant used in the oil industry, using TPA as a substrate (Figure 5) [127]. First, a catechol-producing strain was constructed by introducing the *aroY* gene encoding PCA decarboxylase from *Enterobacter cloacae* into the PCA-producing strain [128]. Next, due to the negative interaction between decarboxylase and hydrolase, a second *E. coli* strain was employed. *E. coli* expressing catechol hydrolases from the *P. stutzeri* OX1 *phKLMNOPQ* gene was developed to produce PYG from catechol [129]. Finally, whole-cell bioconversion employing two engineered strains produced 0.6 mM of PYG from TPA in 12 h with a molar yield of 20%. The PCA-producing strain was also utilized to develop a whole-cell bioconversion system for the production of VA, a direct precursor to vanillin in the food and pharmaceutical industries, using TPA as a substrate (Figure 5) [130]. Initially, an engineered *E. coli* strain capable of producing VA from PCA was developed by introducing Homo sapiens O-methyltransferase (OMT). To enhance PCA conversion to VA, the protein solubility of OMT was enhanced by attaching the hexameric histidine at the N-terminus. This is known to be effective in increasing protein solubility. Moreover, as adenosyl and methyl groups are required for O-methylation by OMT, methionine and glycerol were supplied together with increasing aeration. Finally, whole-cell bioconversion using both PCA- and VA-producing strains produced 1.4 mM of VA from TPA in 48 h with a molar yield of 41.6%.

In another study, the *P. putida* KT2440 strain was engineered to produce β-ketoadipate (BKA), a building block for bio-based polymers, using TPA (Figure 5) [131,132]. The PCA production pathway from TPA was first constructed in the *P. putida* KT2440 strain, as this strain inherently possesses all of the necessary genes for BKA synthesis from PCA. To establish the PCA production pathway, the *tphA2IIA3IIBIIA1II* operon and the *tpaK* gene, responsible for encoding the *Comamonas* sp. E6 TPA dioxygenase, diol dehydrogenase, and *R. jostii* TPA transporter, respectively, were introduced into the chromosome in *P. putida* KT2440, under the control of a strong *tac* promoter. Furthermore, the native *pcaIJ* gene, encoding 3-oxoadipate CoA-transferase, was deleted to prevent undesirable BKA metabolism. Finally, fed-batch fermentation using the final engineered strain produced 15.1 g/L of BKA with a molar yield of 76%.

An alternative approach for the bio-upcycling of TPA towards the production of value-added chemicals involves the utilization of the benzoyl-CoA degradation pathway (Figure 5) [133]. In this pathway, TPA is initially converted to benzoyl-CoA by TPA decarboxylase and benzoic acid CoA ligase. Subsequently, benzoyl-CoA enters the degradation pathway to produce 3-hydroxypimelyl-CoA. The next step involves 3-hydroxyacyl-CoA dehydrogenase, which converts 3-hydroxypimelyl-CoA to crotonyl-CoA. Crotonyl-CoA is further transformed into acetoacetyl-CoA through the sequential actions of crotonyl-CoA hydratase and β-hydroxy butyryl-CoA dehydrogenase. Finally, acetoacetyl-CoA is cleaved into two acetyl-CoA molecules by acetoacetyl-CoA thiolase. While there have not been any previous reports on the production of value-added chemicals through the benzoyl-CoA degradation pathway, it is worth noting that products involving acetyl-CoA as an intermediate have the potential to be explored as potential targets for production from TPA utilizing this benzoyl-CoA degradation pathway.

## 4. Future Perspectives on the Development of Plastic Waste Bio-Upcycling Technology

Bio-upcycling has attracted global recognition as a promising method to address the plastic waste crisis [134]. However, despite ongoing efforts to identify microorganisms and enzymes involved in the degradation and bio-upcycling of plastic waste, the commercialization of this technology has not yet reached the desired level of efficiency. To overcome this challenge, a coordinated approach involving multiple enzymes or microorganisms is necessary, with systems metabolic engineering taking a central role in the development of high-performance strains for plastic degradation and bio-upcycling.

For the first step, recruiting a reliable microbial host strain is foremost to ensure the efficient degradation and bio-upcycling of plastic waste for desired chemical production. The selection of a host strain should take into account the following criteria: (1) possession of metabolic characteristics that make it suitable and applicable for the decomposition and bio-upcycling of plastic; (2) availability of gene and genome engineering tools for efficient manipulation and optimization of the host strain; (3) tolerance toward plastic waste, plastic degradation products, and the target product; and (4) non-pathogenicity to ensure safety in handling and practical application.

The metabolic pathway of the host strain can be reconstructed to enhance both the efficiency of plastic waste degradation and bio-upcycling, as well as to broaden the range of target chemicals of interest. This encompasses strengthening or deleting the native pathway, introducing a heterologous pathway, or creating an entirely new pathway. Recent advancements in the availability of genetic information, metabolism, and enzyme databases, including KEGG [135], MetaCyc [136], and BRENDA [137], respectively, together with pathway prediction tools such as rePrime [138], RetroPath2.0 [139], and RetroRules [140], have greatly facilitated and expedited the metabolic pathway reconstruction process. Alongside the rational metabolic engineering approach, ALE can serve as a robust tool for increasing microbial plastic waste degradation, while simultaneously improving the titer, yield, and productivity of target chemicals of interest [141].

Designing new enzymes through protein engineering or de novo enzyme design can be a powerful strategy to further enhance the efficiency of plastic waste degradation and bio-upcycling. Computational tools that predict ligand binding sites, substrate specificity, protein solubility, and mutational effects on protein function and stability have long served as essential tools in guiding protein engineering strategies [142]. Furthermore, recent trends incorporate artificial intelligence (AI) to steer directed evolution, minimizing the number of experimental iterations needed to attain proteins with the desired properties [143]. This is achieved by leveraging AI to learn from the characteristics of previously characterized variants during screening, enabling the selection of new sequences more likely to exhibit the desired functions. In recent studies, state-of-the-art neural network architectures (e.g., AlphaFold2 and RoseTTAFold) capable of predicting protein structures from amino acid sequences have also shown great potential in elevating the chances of successfully designing de novo enzymes [144]. These AI-aided computational methods will be utilized to provide highly efficient and selective enzymes capable of utilizing recalcitrant plastic wastes in the near future.

## 5. Conclusions

Although the use of plastics is widespread and cost-effective, the prevailing linear plastic lifecycle significantly contributes to environmental pollution and resource depletion in our current plastic era. This review delves into the realm of plastic-degrading enzymes and their associated microorganisms. While significant research efforts have been devoted to PET degradation, with PETase undergoing extensive engineering, other major plastics such as PE, PP, PVC, PU, and PS lack specific degrading enzymes identified thus far. In addition, this review explores the value-added products obtained from bio-upcycling degraded plastic products, considering the framework of systems metabolic engineering. While the substantial challenges that need to be addressed to achieve industrial-level efficiency are acknowledged, the rapid advancement of available tools in this field underscores the expectation that the incorporation of systems metabolic engineering in plastic degradation and bio-upcycling will offer a fundamental solution for addressing the treatment of plastic waste.

## Figures and Tables

**Table 1:** Plastic-degrading enzymes with the highest degradation performance.

Enzyme ^1^	Microorganism	Localization	Degradation Condition ^2^	Results	Ref.
**Polyethylene and Polypropylene**					
Cutinase	*Thermobifida fusca* WSH03-11	Extracellular	60 °C for 7 days using pretreated PE films	Formation of C=O bond Formation of O–H bond	[14]
Lipase	*Halomonas* sp.	Extracellular	30 °C for 10 days using LDPE films	*Halomonas* sp. degraded LDPE, while lipase was identified to take part in LDPE degradation	[15]
Esterase	*Exiguobacterium* sp. *Halomonas* sp. *Ochrobactrum* sp.	Extracellular Extracellular Extracellular	30 °C for 24 h using PE films	Change in surface morphology Formation of surface cracks and deep holes	[16]
P450	*Bacillus thuringiensis* JNU01	Cytoplasm	37 °C for 18 h using PE powder	Formation of C=O bond Formation of O–H bond Formation of amide bond	[10]
Alkane monooxygenase	*Pseudomonas* *aeruginosa* E7	Membrane-bound	37 °C for 80 days using low-molecular-weight PE powder	*E. coli* overexpressing alkB enzyme degraded 18.5% of PE powder	[17]
Laccase	*Botrytis aclada* *Bacillus subtilis*	Extracellular Membrane-bound	30 °C for 5 days using mediator and pretreated PE films	Formation of C=O bond Formation of O–H bond Produced medium-chain-length alcohols and ketones Change in surface morphology	[18]
**Polyurethane**					
Laccase	*Trametes versicolor*	Extracellular	37 °C for 18 days using PCL-PU cubes and coatings	12.1% weight loss in PU cubes 17.2% weight loss in PU coatings	[19]
Cutinase (TfCut2)	*Thermobifida fusca* KW3	Extracellular	70 °C for 200 h using PU cubes	1.9% weight loss in PU cubes Formation of C=O bond Reduction of C–O bond	[20]
Esterase	*Comamonas* *acidovorans* TB-35	Extracellular	30 °C for 24 h using PU cubes	1.2 mg degradation of PU cubes Produced diethylene glycol and adipic acid	[21]
**Polyethylene terephthalate**					
PETase	*Ideonella sakaiensis* 201-F6	Extracellular	37 °C for 72 h using PET films	Produced 140 μM of MHET and TPA	[22]
Cutinase (LCC^WCCG^)	Leaf-branch compost metagenome	Extracellular	72 °C for 10.5 h using pretreated PET	90% degradation of PET	[23]
Cutinase (PE-H^Y250S^)	*Pseudomonas* *aestusnigri* VGXO14T	Extracellular	30 °C for 48 h using PET films	Produced 5.4 ± 0.6 mg/L of MHET	[24]
Cutinase (TfCa^WA^)	*Thermobifida fusca* KW3	Extracellular	50 °C for 1 h using 1 mM of cyclic PET trimer	Produced 45 μM of EMT (95%), MHET (3%), and BHET (2%)	[25]
BHETase (ΔBsEst)	*Bacillus subtilis* PET-86	Extracellular	30 °C for 3 h using 5 mM of BHET	100% conversion of BHET into MHET and TPA	[26]
BHETase (ΔChryBHETase)	*Chryseobacterium* sp. PET-29	Extracellular	30 °C for 9 h using 5 mM of BHET	100% conversion of BHET	[26]
MHETase (MHETase^R411K/S416A/F424I^)	*Ideonella sakaiensis* 201-F6	Extracellular	30 °C for 1 h using 2.5 μM of PET pentamer	100% degradation of PET pentamer	[27]
**Polystyrene**					
Lipase and esterase	*Pseudomonas* spp. *Bacillus* spp.	Extracellular	Treated with culture supernatant, 30 °C for 4 days, using 2 mg/mL of emulsified HIPS	97.4% decrease in turbidity 92.6% decrease in turbidity	[28]
Hydroquinone peroxidase	*Azotobacter beijerinckii* HM121	Extracellular	30 °C for 10 min using 2 g/L of PS	Reduction in the average molecular weight of PS	[29]
**Polylactic acid**					
PLA depolymerase	*Paenibacillus* *amylolyticus* strain TB-13	Extracellular	37 °C for 6 h using 0.5% of emulsified PLA	100% decrease in turbidity	[30]
Proteinase K	*Tritirachium album*	Extracellular	37 °C for 6 h using 0.5% of emulsified PLA	61.5% decrease in turbidity	[30]
Lipase	*Pseudomonas* sp. strain DS04-T	Extracellular	50 °C for 3 h using 1 g/L of emulsified PLA	Produced lactic acid monomers Decrease in turbidity	[31]
**Polybutylene succinate**					
Cutinase	*Fusarium solani*	Extracellular	37 °C for 26 h, pretreated PBS films	100% degradation of PBS Produced succinic acid, butanediol-succinate-butanediol, and succinate-butanediol-succinate Formation of surface cracks	[32]
Lipase (Lipase Asahi)	*Chromobacterium* *viscosum*	Extracellular	37 °C for 17 days using PBS films	100% degradation of PBS	[33]
Lipase (Lipase PS^®^)	*Pseudomonas cepacia*	Extracellular	50 °C for 7 days using PBS films	Produced 4HBS	[34]

^1^ Engineered enzymes are indicated by the name in parentheses. ^2^ The degradation conditions refer to either the enzyme treatment or the cell culture condition. Abbreviations: PE, polyethylene; PP, polypropylene; LDPE, low-density polyethylene; PU, polyurethane; PCL, polycaprolactone; PET, polyethylene terephthalate; MHET, mono(2-hydroxyethyl) terephthalate; TPA, terephthalic acid; EMT, 1,2-ethylene-mono-terephthalate-mono(2-hydroxyethyl) terephthalate; BHET, bis(2-hydroxyethyl) terephthalate; PS, polystyrene; HIPS, high impact polystyrene; PLA, polylactic acid, PBS, polybutylene succinate; 4HBS, 4-hydroxybutyl succinic acid.

**Table 2:** Plastic-degrading microorganisms with the highest degradation performance.

Microorganism	Degradation Condition	Results	Ref.
**Polyethylene and Polypropylene**			
*Pseudomonas aeruginosa* WGH-6	Modified mineral salt medium containing PP particles at 30 °C for 40 days	17.2 ± 1.56% weight loss of PP particles Formation of C=O bond WCA decreased from 108.22° to 56.88°	[35]
*Microbacterium paraoxydans*	Minimal broth containing 0.25 g of pretreated LDPE powder at room temperature for 2 months	Utilized PE as a sole carbon source 61% weight loss in LDPE powder	[36]
*Pseudomonas aeruginosa*	Minimal medium containing 0.25 g of pretreated LDPE powder at RT for 2 months	50.5% weight loss in LDPE powder	[36]
*Alternaria alternata* FB1	Basic medium containing PE films for 28 days	Formation of C=O bond Produced diglycolamine Crystallinity decreased from 62.79% to 52.02%	[37]
*Aneurinibacillus* spp. *Brevibacillus* spp.	Minimal medium containing LDPE, HDPE, and PP films and pellets at 50 °C for 140 days	45.7% weight loss in LDPE films 37.2% weight loss in HDPE films 44.2% weight loss in PP films Formation of C=O bond Produced tridecanoic acid and octadecanoic acid Formation of biofilm	[38]
*Pseudomonas Vibrio* *Aspergillus niger*	B7 medium supplemented with starch containing PE strips at 30 °C for 175 days	40% weight loss in PE strips Formation of C=O bond Formation of O–H bond Produced hydrocarbon mixtures (C_10_H_22_ to C_31_H_64_)	[39]
**Polyvinyl chloride**			
*Klebsiella* sp. EMBL-1	Mineral salt medium containing PVC films at 30 °C for 90 days	Utilized PVC as a sole carbon source 19.57% weight loss in PVC films Formation of C=C bond Formation of O–H bond Produced hydrocarbon mixtures (C_20_H_42_, C_21_H_44_, C_24_H_50_, and C_25_H_52_)	[40]
**Polyurethane**			
*Comamonas acidovorans*	Basal medium containing PU cubes at 30 °C for 7 days	100% weight loss in PU cubes Produced diethylene glycol, trimethylolpropane, and adipic acid	[41]
*Rhodococcus equi* TB-60	Mineral medium containing TDCB at 30 °C for 10 days	70% degradation of TDCB Produced 2,4-TDA	[42]
Polyethylene terephthalate			
*Ideonella sakaiensis* 201-F6	Yeast extract–sodium carbonate– vitamins medium containing PET films at 30 °C for 13 days	10.7 mg degradation of PET films Produced MHET, BHET, and TPA Change in surface morphology	[43]
**Polystyrene**			
*Pseudomonas aeruginosa* DSM 50071	Liquid carbon-free basal medium containing PS films at 25 °C for 60 days	Utilized PS as a sole carbon source Formation of O–H bond Formation of C=O bond Reduction of C–C bond Formation of holes and edge smoothing WCA decreased from 91.56° to 79.8°	[44]
*Acinetobacter johnsoniii* JNU01	Basal medium containing PS powder at 28 °C for 7 days	Utilized PS as a sole carbon source Formation of O–H bond Change in surface morphology WCA decreased from 84.29° to 66.03°	[45]
*Bacillus paralicheniformis* G1	Mineral salt medium containing PS films at 30 °C for 60 days	Utilized PS as a sole carbon source 34% weight loss in PS films	[46]
**Polylactic acid**			
*Saccharothrix waywayandensis*	Basal medium supplemented with 0.1% gelatin containing 100 mg of PLA films at 30 °C for 7 days	95% weight loss in PLA films Change in surface morphology Formation of holes	[47]
*Amycolatopsis* HT-32	Basal medium containing 100 mg of pretreated PLA films at 30 °C for 14 days	60% weight loss in PLA films Change in surface morphology Formation of semi-spherical holes	[48]
*Tritirachium album* ATCC 22563	Basal medium supplemented with 0.1% gelatin containing 100 mg of PLA films at 30 °C for 14 days	76.4 ± 2.5% weight loss in PLA films Produced 14.2 mg of lactic acid	[49]
*Kibdelosporangium aridum*	Basal medium supplemented with 0.1% gelatin containing 100 mg of high-molecular-weight PLA films at 30 °C for 10 days	81% weight loss in PLA films Change in surface morphology Formation of pits	[50]

Abbreviations: WCA, water contact angle; HDPE, high-density polyethylene; PVC, polyvinyl chloride; TDCB, toluene-2,4-dicarbamic acid dibutyl ester; 2,4-TDA; 2,4-toluenediamine.
